# The Role of Senescence-Associated Secretory Phenotype in Bone Loss

**DOI:** 10.3389/fcell.2022.841612

**Published:** 2022-02-09

**Authors:** Runjiu Zhu, Haoyang Wan, Hong Yang, Mingrui Song, Yu Chai, Bin Yu

**Affiliations:** ^1^ Department of Orthopaedics, Nanfang Hospital, Southern Medical University, Guangzhou, China; ^2^ Guangdong Provincial Key Laboratory of Bone and Cartilage Regenerative Medicine, Nanfang Hospital, Southern Medical University, Guangzhou, China; ^3^ Department of Respiratory and Critical Care Medicine, Nanfang Hospital, Southern Medical University, Guangzhou, China

**Keywords:** senescence, senescence-associated secretory phenotype (SASP), osteoporosis, bone loss, stem cell

## Abstract

As the population of most nations have a large proportion of older individuals, there is an increase in the prevalence of osteoporosis. Consequently, scientists have focused their attention on the pathogenic mechanisms of osteoporosis. Owing to an increase in studies on cellular senescence in recent years, research has begun to focus on the function of the senescent microenvironment in osteoporosis. With chronic inflammation, senescent cells in the bone marrow secrete a series of factors known as senescence-associated secretory phenotype (SASP) factors, acting on their own or surrounding healthy cells and consequently exacerbating ageing.The components of the SASP may differ depending on the cause of osteoporosis. This review aimed to summarize the relationship between SASP factors and osteoporosis and suggest new insights into the mechanistic investigation of osteoporosis.

## Introduction

Osteoporosis is a systemic skeletal condition characterized by decreased bone mass and degeneration of the bone tissue microstructure. Osteoporosis-related fractures are becoming more prevalent in the elderly and result in a series of problems and a higher mortality risk. There is a strong association between ageing and osteoporosis (Li et al., 2021). New approaches to osteoporosis treatment have resulted from research on the fundamental mechanisms of bone resorption and production (Compston et al., 2019). Cellular senescence was first proposed by Hayflick and Moorhead (1961) (Hayflick and Moorhead 1961) and is described by cell cycle arrest. Cellular senescence occurs when cells lose the ability to proliferate and differentiate over time or in response to external stresses (Lopez-Otin et al., 2013). Cell death due to senescence and growth of new cells in living organisms are in a dynamic balance (Perez-Figueroa et al., 2021). The connection between cellular senescence and chronic disease is associated with a variety of chronic diseases such as atherosclerosis, diabetes, and osteoporosis (Stojanovic et al., 2020; Crespo-Garcia et al., 2021; Deng et al., 2021)). The concept of the senescence-associated secretory phenotype (SASP) was first introduced by Jean-Philippe Copper in 2008 for research on human malignancies. This research demonstrated that senescent cells can promote precancerous cells to become cancerous by secreting some substances (Coppe et al., 2008). These substances were defined as SASP factors (Coppe et al., 2008). In recent years, the role of SASP factors in the disease microenvironment has been emphasized in the study of tumors and chronic inflammatory diseases (Acosta et al., 2013). SASP has detrimental paracrine and systemic effects in chronic inflammation, including the induction of senescence in healthy cells (Coppe et al., 2008). By contrast, recent research has demonstrated that senescent human dermal fibroblasts accelerate the healing of keratinocyte scratches and stimulate fibroblast differentiation (Hou and Kim 2018). The SASP factors can also recruit and activate immune cells during tumour development (Acosta et al., 2013).

## Senescence-Associated Secretory Phenotype Functional Classification

The SASP affects cellular interactions *in vivo* and is inextricably linked to cellular senescence, ageing, and age-related diseases (Hubackova et al., 2012; Zhao et al., 2021). The SASP factors can activate the body’s immune system (Jin et al., 2021). This activation may promote the repair of damaged tissues or contribute to a chronic inflammatory response (Jin et al., 2021). Chronic inflammation is associated with many age-related diseases, such as cardiovascular diseases (Ritschka et al., 2017; Ferrucci and Fabbri 2018). The composition of SASP depends on the cell type and the nature of the initial stimulus (Di Micco et al., 2021). Although the core components remain similar, there are differences in the quality and quantity of the SASP in different tissues and ageing models (Aquino-Martinez et al., 2020; Di Micco et al., 2021). The SASP is composed of a series of proinflammatory factors, chemokines, growth factors and proteases, which is produced by stimulation of multiple factors *in vivo* and *ex vivo* when cells become senescent (Ortiz-Montero et al., 2017; Wang et al., 2019; Basisty et al., 2020; Kawagoe et al., 2020). These *in vivo* and *ex vivo* factors include tumour necrosis factor-α(TNF-α), interleukin (IL)-6, IL-1, IL-8, matrix metalloproteinase (MMP), granulocyte colony-stimulating factor (G-CSF) and plasminogen activator inhibitor-1 (PAI-1) (Coppe et al., 2008; Lim et al., 2015; Maciel-Baron et al., 2016; Oubaha et al., 2016; Ruscetti et al., 2018; You et al., 2019) (Table 1). Depending on the type and function of the SASP components, they can be divided into the following categories: Table 1

**TABLE 1:** Classification and functional list of senescence-associated secretory phenotype.

Classification	Name	Function	References
Interleukin	IL-1α	Inhibit B lymphocyte formation	Behnia et al. (2015); Wiley et al. (2016)
	IL-1β	Promote inflammation and induce stem cell senescence	Behnia et al. (2015); Ortiz-Montero et al. (2017); Martini et al. (2019)
	IL-6	Associated with tumor cell invasion	Orjalo et al. (2009); Behnia et al. (2015)
	IL-7	Regulate B lymphocyte production and maintain BMSC function	Stephan et al. (1998); Hou et al. (2019)
	IL-15	Activate natural killer cells and remove senescent cells	Hou et al. (2019); Schafer et al. (2020)
Chemokines	CCL27	Reduces immune cell function	Andriani et al. (2016); Degos et al. (2019)
	IL-8	Increased tumor cell invasion	Orjalo et al. (2009); Behnia et al. (2015)
	MIP-3a	Recruitment of inflammatory cells	Matsui et al. (2001); Behnia et al. (2015)
	GRO	Promote tumorigenesis	Yang et al. (2006); Hou et al. (2019)
	ENA-78	Regulates angiogenic activity	Keane et al. (2001); Behnia et al. (2015)
Growth Factor	AREG	Maintain immune cell function	Hou et al. (2019); Xu et al. (2019)
	EGF	Regulating cell proliferation	Salehinejad et al. (2013); Behnia et al. (2015)
	VEGF	Regulate angiogenesis	Freudenberg et al. (2015); Marazita et al. (2016); Hou et al. (2019)
	HGF	Maintenance stem cell characteristics	Cao et al. (2020); Rohn et al. (2020)
	IGFBP-4	Accelerated cell senescence	Hou et al. (2019); Alessio et al. (2020)
	IGFBP-6	Retards cell senescence	Hou et al. (2019); Xu et al. (2021)
	IGFBP-7	Accelerated cell senescence	Severino et al. (2013)
Matrix Metalloprotein-ase	MMP-1	Accelerated osteogenic differentiation of BMSC	Behnia et al. (2015); Wu et al. (2020)
	MMP-3	Degradation of extracellular matrix	Behnia et al. (2015); Niwa et al. (2020)
	MMP-9	Degradation of extracellular matrix	Behnia et al. (2015); Hilliard et al. (2020)
	MMP-13	Regulates tumor angiogenesis	Li et al. (2017); Bao and Hu (2018); Gao et al. (2018)
	TIMP-1	Inhibit extracellular matrix degradation	Yokose et al. (2012); Behnia et al. (2015)

## The Biological Role of Senescence-Associated Secretory Phenotype

The SASP can have both positive and negative effects on an organism (Ritschka et al., 2017). Cellular senescence and the SASP can repair cells, restore tissue integrity, and promote wound healing (Demaria et al., 2014). It has also been shown that cellular senescence inhibits tumor growth by inhibiting cell proliferation and differentiation (Acosta et al., 2008). However, senescent cells do not lose their ability to interact with other cells and can secrete factors that activate the immune system (Mosteiro et al., 2016). Senescent cells can recruit large numbers of immune cells such as macrophages and natural killer cells to remove senescent cells (Mosteiro et al., 2016). Simultaneously, senescent cells can release cytokines that transmit senescence signals to surrounding cells to inhibit the proliferation of senescent cells (Hubackova et al., 2012). SASP factor release occurs due to the DNA damage response triggered by external stimuli (Slawinska and Krupa 2021). Recently, research has focused on the role of SASP in chronic degenerative diseases, such as neurodegenerative lesions, osteoarthritis, and osteoporosis (Faust et al., 2020; Sharma et al., 2020; Gaikwad et al., 2021).

## The Role of Senescence-Associated Secretory Phenotype in Bone Loss

### Senescence-Associated Secretory Phenotype in the Bone Marrow Cavity

**FIGURE 1:**
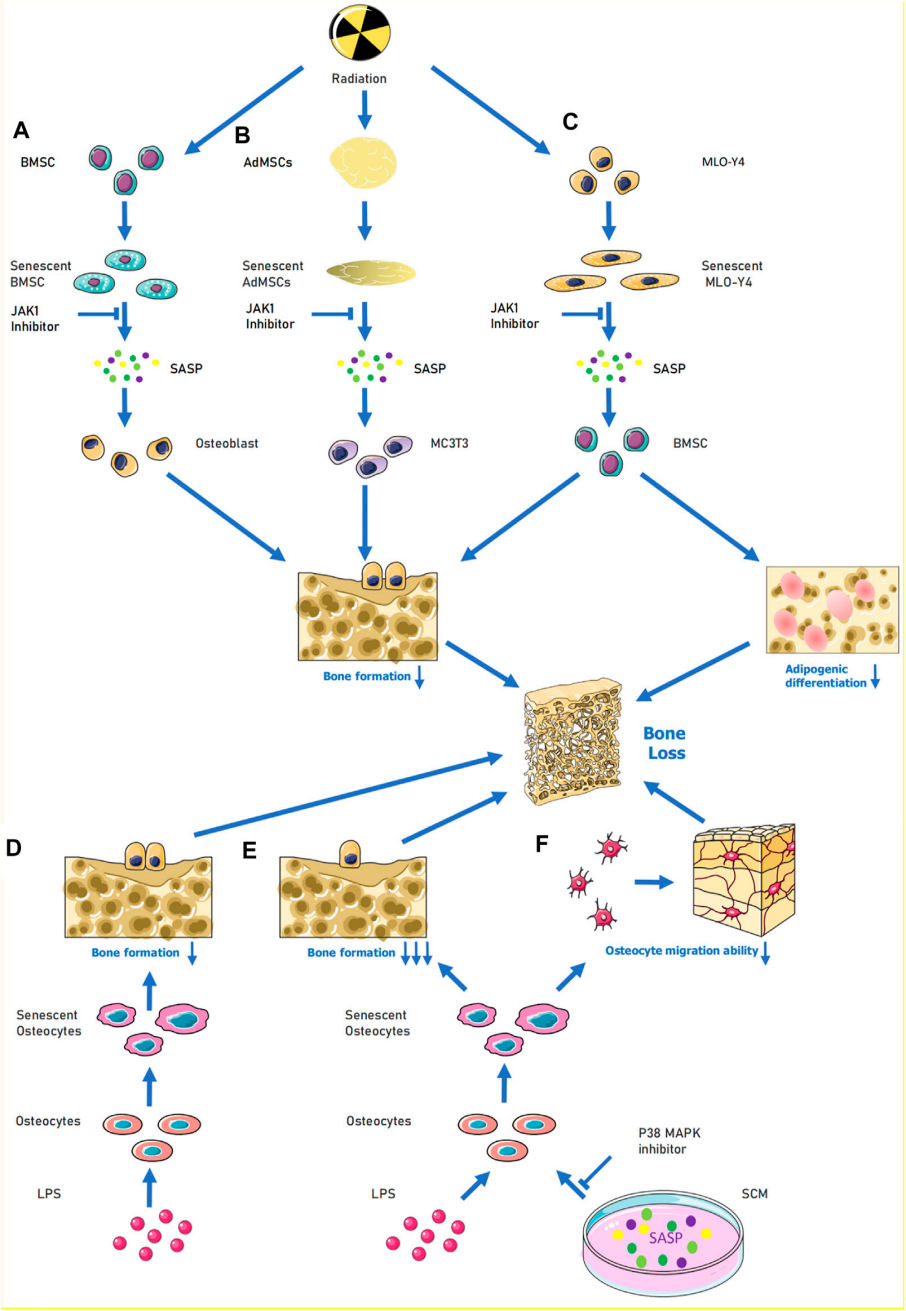
When cells are senescence induced by external stimulation, SASP can be released to aggravate their own senescence or induce the senescence of surrounding normal cells. For **(A)**, under the influence of radiation, BMSCs become senescent, causing activation of the JAK1/STAT3 pathway and release of SASP (e.g., IL-6, IL-8, MMP9) to reduce the osteogenic differentiation ability of osteoblasts. **(B)** When MLO-Y4 cells were irradiated, MLO-Y4 was induced to senesce, which in turn released SASP (e.g,. IL-1α, IL-6, MMP-3, IGFBP-6, etc.) thus affecting the normal growth of BMSC and reducing their osteogenic differentiation ability and adipogenic differentiation ability. In **(C)**, human adipose MSCs were induced to undergo senescence by radiation, and the released SASP could inhibit MC3T3 osteogenic differentiation, and the process could be alleviated by JAK inhibitors. **(D–F)**, LPS acting on osteoblasts induced senescence and thus inhibited osteogenic differentiation of osteoblasts; when LPS and SCM (senescence conditioned medium) were combined to act on osteoblasts, the osteogenic differentiation and migration ability of osteoblasts were greatly reduced, and this process could be alleviated by P38-MAPK inhibitor. Abbreviations: SCM, senescence-conditioned mediators, LPS, Lipopolysaccharide.

In the field of age-related osteoporosis research, the study of SASP in the bone marrow senescent microenvironment is still in its early stages. The presence of senescent cells and their release of SASP factors were demonstrated in an age-related osteoporosis mouse model (Farr et al., 2016). This study extracted cells from the marrow cavities of young and old mice and demonstrated that several SASP factor mRNA levels were increased in osteoblasts (e.g., *Mmp12, Mmp3*, etc.). Given that osteocytes are key factors in bone remodeling, the role of senescent osteocytes and their production of SASP factors may help explain the pathogenesis of age-related bone loss (Farr et al., 2016). Evidence suggests that age-related bone loss can be attenuated by eliminating senescent cells from the bone marrow microenvironment *in vivo*. *In vitro* experiments have shown that conditioned media produced by senescent cells suppress osteoblast mineralization and that this process can be alleviated by JAK inhibitors (Figure 1A). These experiments suggest that cellular senescence and the release of SASP factors may play key roles in age-related osteoporosis (Farr et al., 2017) (Figure 1B). Radiation-induced bone loss has received increasing attention in recent years and some scholars have investigated specific cells that are associated with the pathways involved. Evidence suggests that radiation causes bone marrow mesenchymal stem cells (BMSCs) to senesce and activates the januskinase 1/signal transducer and activator of transcription 3 pathway in these cells, which, in turn, secrete SASP factors. The conditioned medium of senescent BMSCs was shown to have a negative effect on osteogenic differentiation. By contrast, the addition of a JAK1 inhibitor to the medium of senescent BMSCs can decrease the senescent cell secretion of negative SASP factors and slow down the adverse effects on osteoblast osteogenic differentiation (Bai et al., 2020). Radiation may also lead to the release of SASP factors from ageing osteoblasts and act on BMSCs to interfere with their osteogenic differentiation. The osteogenic differentiation potential of BMSCs is affected by the release of SASP factors through the paracrine pathway when ageing murine long bone osteocyte Y4 (MLO-Y4) cells are induced by radiation (Xu et al., 2021) (Figure 1C). However, treatment of ageing MLO-Y4 cells with a JAK1 pathway inhibitor blocks the secretion of SASP factors and partially alleviates the inhibition of osteogenic differentiation of BMSCs (Xu et al., 2021). Radiation can also cause the ageing of osteoblasts and BMSCs and lead to the secretion of SASP factors. These SASP factors can then affect osteoblast and BMSC osteogenic differentiation. The effect of the toxic heavy metal cadmium on BMSCs has recently been investigated. Cadmium induced senescence in BMSCs by upregulating the NF-κB signalling pathway, and these cells subsequently released SASP factors. It was also demonstrated that cadmium exposure delayed bone repair and regeneration after cranial defect surgery. This research elucidates the role and mechanism of cadmium in osteoporosis, and it could lead to a new treatment option for cadmium-related bone loss. (Luo et al., 2021). Studies have also demonstrated an association between obesity, ageing, and abnormal skeletal development in offspring. During early maternal pregnancy, maternal obesity can lead to abnormal foetal and postnatal skeletal development. High fat diet-induced maternal obesity reduced foetal skeletal development and enhanced foetal osteoblast senescence signaling. In the bone progenitors of the offspring of pregnant obese, senescent bone progenitors released SASP factors This may be explained by the epigenetic regulation (*via* histone acetylation) of the genes involved in senescence signalling in developing foetal osteoblasts (Chen et al., 2018).

### Senescence-Associated Secretory Phenotype in Periodontal Tissue

Periodontitis is characterized by chronic inflammation of periodontal supporting tissues and can lead to bone loss in the teeth when inflammation occurs in the alveolar bone and jaws. In a model of hyperglycaemia-induced periodontitis, bone loss has been shown to be associated with ageing. A transgenic diabetic model has demonstrated that periodontal senescence in young diabetic mice is accompanied by the accumulation of senescent macrophages and enhanced early macrophage SASP responses. GLUT1 sensors are important for hyperglycaemia-induced macrophage senescence and SASP responses. Hyperglycaemia-induced macrophage senescence releases SASP factors into other tissues in the periodontium to induce immune responses. This may highlight a potential molecular mechanism of bone loss in diabetic periodontitis (Wang et al., 2021). Periodontitis can also occur due to a progressive change from commensal to pathogenic oral flora. Bacterial-derived lipopolysaccharide (LPS) induces the accumulation of senescent osteoblasts in the alveolar bone of young mice and leads to upregulation of genes (*Icam1*, *Il6*, *Il17*, *Mmp13,* and *Tnfα*) involved in SASP. The secretion of SASP factors promotes the proliferation of certain oral bacteria, which, in turn, produce more LPS (Aquino-Martinez et al., 2020). This exacerbates the senescence of alveolar bone cells and may lead to alveolar bone loss (Aquino-Martinez et al., 2020). In age-related alveolar bone loss, the accumulation of senescent bone cells contributes to the deterioration of the periodontal environment by exacerbating chronic inflammation and reducing the regeneration of older bone cells. Moreover, cellular senescence can enhance the inflammation induced by bacterial components. In osteoblasts, IL6, IL17, IGFBP4, and MMP13 levels are significantly higher with age. *In vitro* senescence-conditioned mediates enhanced LPS-induced expression of IL1α, IL1β, and IL6 in osteoblasts. This, in turn, affects cell migration and osteogenic differentiation These *in vitro* effects were partially ameliorated by the p38 mitogen-activated protein kinase (MAPK) inhibitor (Aquino-Martinez et al., 2021) (Figure 1D–F). Melatonin protects osteoblasts from ethanol-induced cellular senescence in human alveolar bone and inhibits osteoclast differentiation. Melatonin blocks the ethanol-induced activation of mammalian target of rapamycin, AMP-activated protein kinase, MAPK, and nuclear factor of activated T cells c-1 pathways. This downregulated the expression of SASP-related genes (including *Il1β*, *Il6*, *Il8*, and *Tnf*) and possibly the secretion of SASP factors, thereby maintaining homeostasis (Bae et al., 2018). This reversed the osteogenic differentiation of the suppressed cells.

Taken together, these studies suggest that cellular senescence and the release of SASP markers by various factors may be key mechanisms leading to senescence-associated bone loss. Therefore, it is important to define the SASP at the proteomic level in senescent cells and to develop ways to slow down the progression of osteoporosis.

## Discussion

In the ageing milieu of the bone marrow cavity, SASP is primarily generated by BMSCs and osteoblasts, with senescent cells secreting the majority of SASP factors in the setting of chronic inflammation. Furthermore, SASP that is released from senescent BMSCs and osteoblasts has an extremely inhibitory effect on bone formation. Cells in the bone marrow cavity include BMSCs, osteoblasts and vascular endothelial cells, etc. However, the relationship between SASP that is released from senescent vascular endothelial cells in the bone marrow lumen and bone loss has been less frequently reported. The link between SASP and vascular endothelial cell senescence is extremely strong in other diseases associated with vascular senescence (Prattichizzo et al., 2016). This suggests that senescent vascular endothelial cells may play an important role in senescence-associated osteoporosis. Overall, researchers are now increasingly interested in the role of SASP in osteoporosis, but most studies have concluded that SASP has an inhibitory effect on bone mass formation. Does SASP contribute to bone mass formation under certain conditions? We don’t know, and perhaps subsequent studies will change our current view of SASP. This review also establishes a framework for future work that can investigate the role of SASP in the bone marrow microenvironment.

## Abbreviations

AREG, amphiregulin; BMSCs, bone marrow mesenchymal stem cells; CCL27 cc motif chemokine ligands; ENA-78 epithelial neutrophil-activating protein 78; EGF epidermal growth factor; GRO growth-regulated oncogene; HGF hepatocyte growth factor; IGFBP insulin-like growth factor binding protein; LPS lipopolysaccharide; MIP-3a macrophage inflammatory protein-3 alpha; MMP matrix metalloproteinase; MLO-Y4, murine long bone osteocyte Y4; SASP, senescence-associated secretory phenotype; TIMP tissue inhibitor of metalloproteinases; VEGF vascular endothelial growth factor.
